# Trends in Psychiatric Day Care Practices in Japan: An Analysis Using the National Database of Health Insurance Claims Open Data

**DOI:** 10.7759/cureus.63166

**Published:** 2024-06-25

**Authors:** Tomoyuki Okazaki, Satoshi Asaoka, Hitoshi Okamura

**Affiliations:** 1 Rehabilitation, Medical Corporation Kouseikai, Kusatsu Hospital, Hiroshima, JPN; 2 Clinical Psychology, Medical Corporation Kouseikai, Kusatsu Hospital, Hiroshima, JPN; 3 Psychiatry, Graduate School of Biomedical and Health Sciences, Hiroshima University, Hiroshima, JPN

**Keywords:** epidemiology, covid-19, ndb open data japan, psychiatric rehabilitation, psychiatric day care

## Abstract

Background: Psychiatric day care is a key framework for outpatient rehabilitation in Japanese psychiatric treatment. It targets a wide range of diseases and performs various roles. The current status and utilization of psychiatric day care in Japan have changed owing to an increase in the availability of other social resources and the coronavirus disease 2019 (COVID-19) pandemic. However, no reports have quantitatively analyzed the trends or current situation of psychiatric day care in Japan. Therefore, this study aimed to analyze the status of the operation of psychiatric day care in Japan and its trends by sex, region, and age group.

Methods: Based on the publicly available data from the Japanese National Database of Health Insurance Claims and Specific Health Checkups (NDB Open Data), we investigated changes in the numbers related to psychiatric day care nationwide from fiscal year (FY) 2017-2021. Furthermore, we calculated the numbers of psychiatric day care facilities use per 1,000 people by prefecture, sex, and age group in 2021. A Mann-Whitney U test was conducted to compare the total number of psychiatric day care facilities used in months during periods of emergency declarations and priority preventative measures with those used in months when there were no such periods. Pearson's correlation coefficient test was conducted to examine the correlation between the numbers of psychiatric beds per 100,000 people and the annual numbers of psychiatric day care facilities use per 1,000 people in each prefecture. A Mann-Whitney U test was conducted to compare the number per 1,000 people per year of prefectures with or without capital- and government-designated cities and compare the annual total number (FY 2017-2021) by sex.

Results: Overall, the number of psychiatric day care facilities use tended to decrease. The monthly data showed a significant decrease in the total number of psychiatric day care facilities’ usage during periods of emergency declarations and priority preventative measures (p=0.02). The numbers of "psychiatric short care" use increased in 2021 and those of psychiatric day-night care (>3 years / >3 days per week) use showed an increasing trend from 2020. The overall number of psychiatric day care facilities’ usage per 1,000 people by prefecture was significantly lower in prefectures having cities with populations of 700,000 or more (p<0.01). A significant positive correlation was found between the number of psychiatric day care facilities use per 1,000 people by prefecture and the number of psychiatric beds per 100,000 people by prefecture (r=0.61, p<0.01). The usage of psychiatric day care facilities was significantly lower for women (p<0.01). In terms of age group, the use of psychiatric day care facilities was significantly lower for younger age groups (under 65 years of age) (p<0.01).

Conclusions: Trends in the numbers reflected differences in user attributes and regional differences. It is necessary to establish a management system tailored to individual attributes and in regions where support is difficult to reach.

## Introduction

Psychiatric day care is a typical framework to provide psychiatric rehabilitation to outpatients in Japan. In the Japanese medical fee system, psychiatric day care is defined as the treatment of patients with mental illnesses using appropriate individual programs to improve social functioning. Psychiatric day care in Japan was established in the 1960s to facilitate a return to community life after hospitalization. Although Japan has a high number of psychiatric beds compared to other countries, psychiatric day care has developed because of stipulations in the reimbursement system and the reduction of psychiatric beds [1]. In 2006, there were more than 1,500 day care facilities in Japan [1].

Present psychiatric day care programs include psychoeducation, cognitive rehabilitation [2], physical activity or sports, handicrafts, creative activities, recreation, interpersonal communication, social skills, and instrumental activities of daily living [3]. Psychiatric day care covers a wide range of disorders, performs various roles, and is classified into place-to-stay-type [4] and active facilities [1]. Place-to-stay facilities are far from community social resources and are mainly used to provide long-term support to patients with chronic schizophrenia. Place-to-stay facilities are for patients with a long history of hospitalization or severe and chronic conditions who require the support of medical staff for relapse prevention, the management of medical conditions, and daily life activities. Active facilities specialize in targets and objectives aimed at returning to society [1]. For example, facilities for young patients are focused on improving daily living, symptom management, and interpersonal skills with a view to employment or schooling [2]. Several reports on the effectiveness of psychiatric day care in Japan have provided evidence of the prevention of rehospitalization in patients with schizophrenia [5,6] and the deterioration of illness, improvement in daily functioning and social skills [7], and contribution to treatment continuity in patients with schizophrenia and bipolar disorder [3,8].

In recent years, the number of social resources, including welfare services for persons with disabilities, has increased as commercial enterprises entered the market since the promulgation of the Comprehensive Support for Persons with Disabilities Act in 2013 [9]. Welfare service facilities provide support to people with disabilities and illnesses to enable them to live in the community. This includes support for employment, independent training facilities, and group homes. These former roles of psychiatric day care facilities have been transferred to welfare service facilities. Additionally, since 2020, the coronavirus disease 2019 (COVID-19) pandemic has brought about many changes in Japanese society. Some psychiatric day care facilities were forced to limit the hours and frequency of operation, number of patients, and activities to conform with stay-at-home restrictions and avoid the three Cs (closed spaces, crowded places, and close-contact settings) [10] for infection control in Japan. A survey of emergency psychiatric facilities indicated that more than 70% of the responding facilities had experienced closures or restrictions of day care activities [11].

Evidently, the status of psychiatric day care in Japan has changed in recent years. However, no reports have quantitatively examined the changes in operation (e.g., the total medical fees) of psychiatric day care in Japan. Owing to the diversity of psychiatric day care, its target user population may differ depending on the facility and region of operation. However, no reports have quantitatively considered the differences in usage by sex, age group, and region in Japan. In other words, the attributes of patients who utilize psychiatric day care facilities (frequently or periodically) have not been quantitatively identified. It is useful to understand the usage of psychiatric day care facilities and patient attributes for the future provision of psychiatric outpatient rehabilitation. Therefore, it is necessary to quantitatively analyze the status and evolution of psychiatric day care facility operations in Japan by sex, prefecture, age group, and year.

The Japanese Ministry of Health, Labour and Welfare (MHLW) has published the annual numbers for each treatment provided “by prefecture,” “by month,” and “by sex and age” in the National Database of Health Insurance Claims and Specific Health Checkups (NDB) as Open Data. Japan has universal health insurance, and the NDB Open Data aggregates domestic receipt information and is comprehensive. Studies using the NDB Open Data have been published [12-14]. An analysis of the NDB Open Data helped us understand the trends and general view of medical care as well as the actual status of psychiatric day care facility utilization in Japan. The aims of this study were (1) to indicate the number of times psychiatric day care facilities have been used in recent years, (2) to clarify the regional differences in psychiatric day care facilities used, and (3) to indicate differences in the number of psychiatric day care facilities used by sex and age group.

## Materials and methods

Data collection

The NDB Open Data [15] contains data on health insurance claims receipts in Japan, making it suitable for analyzing the actual conditions and trends in medical care. The latest data are from fiscal year (FY) 2021; this study used data from FY 2017-2021. Based on data from medical inpatient/outpatient receipts and Diagnosis Procedure Combination receipts, the number of claims for each medical practice for each medical fee point was aggregated by 47 prefectures in Japan, sex, five-year age group, and month. Medical practice code numbers used in this study are shown in Table 1. Items which aggregated to less than 10 were not publicly available and were counted as zero in this study. The medical practice items for psychiatric day care facilities in Japan are classified into short (3 h/day), day (6 h/day), day-night (10 h/day), and night care (after 4 p.m. and 4 h/day), depending on the length of use. If a patient has used day, day-night, and night care services for more than three days per week for three years after the date of initial use, the services were categorized as >3 years / >3 days per week. Psychiatric short and day care were categorized as large or small based on the number of persons available per day, staffing, and size of the facility area. This study used items related to short, day, day-night, and night care for the medical practices. Items related to inpatients were excluded. The total number of psychiatric day care facilities used was calculated and used as the sum of all medical practices related to psychiatric day care facilities.

**Table 1:** Medical practice code numbers used in this study

Medical practice	Code number	Length of use
Psychiatric short care (small)	180028610	3 hours
Psychiatric short care (large)	180028710	3 hours
Psychiatric day care (small)	180007510	6 hours
Psychiatric day care (large)	180007610	6 hours
Psychiatric day care (>3year / >3days per week) (small)	180048030	6 hours
Psychiatric day care (>3year / >3days per week) (large)	180048130	6 hours
Psychiatric night care	180007810	After 4 p.m. and 4 hours
Psychiatric night care (>3year / >3days per week)	180048430	After 4 p.m. and 4 hours
Psychiatric day-night care	180017210	10 hours
Psychiatric day-night care (>3year / >3days per week)	180048530	10 hours

Analysis

Changes by Fiscal Year

We used the annual total number of each medical practice. The total of all items listed in Table 2 was calculated as “The number of psychiatric day care facilities used.” The values for psychiatric short and day care were calculated and used as the sum of large and small values, respectively. To examine the impact of COVID-19, monthly data for FY 2020-2021 were used. A Shapiro-Wilk’s test was performed to check that the data did not follow a normal distribution. A Mann-Whitney U test was conducted to compare the total number of psychiatric day care facilities used (the total value of items are presented in Table 2) in months during periods of emergency declarations (April-May 2020 and January-March 2021, April-June 2021, July-September 2021) and priority preventative measures (April-September 2021, January-March 2022) (N=14), with those used in months when there were no such periods (N=10).

**Table 2:** The annual numbers of use by fiscal year (FY2017 - 2021) (times) a: The sum of “Psychiatric short care (small)” and “Psychiatric short care (large)” b: The sum of “Psychiatric day care (small)” and “Psychiatric day care (large)” c: The sum of “Psychiatric day care（>3year / >3days per week）(small)” and “Psychiatric day care（>3year / >3days per week）(large)” *: The sum of a + b + c + d + e + f + g

Medical practice	FY2017	FY2018	FY2019	FY2020	FY2021
Total number of psychiatric day care facilities use*	8664214	8554146	8395732	7553463	7620183
Psychiatric short care (small + large)^a^	1104848	1129986	1163177	1093189	1172482
Psychiatric short care (small）	296802	310278	319498	281554	300132
Psychiatric short care (large)	808046	819708	843679	811635	872350
Psychiatric day care (small + large)^b^	5576011	5473258	5326099	4667436	4653033
Psychiatric day care (small)	968627	919113	894628	746877	756154
Psychiatric day care (large)	4607384	4554145	4431471	3920559	3896879
Psychiatric day care（>3year / >3days per week） (small + large)^c^	262875	262927	261889	237486	238366
Psychiatric day care（>3year / >3days per week）（small）	38206	39122	37418	31313	32749
Psychiatric day care（>3year / >3days per week）（large）	224669	223805	224471	206173	205617
Psychiatric night care^d^	93577	96400	91082	82809	87128
Psychiatric night care (>3year / >3days per week)^e^	6356	6254	5544	5875	6150
Psychiatric day-night care^f^	1276573	1241405	1207087	1148750	1142360
Psychiatric day-night care（>3year / >3days per week)^g^	81099	80989	78965	80432	82298

Regional Difference

Region refers to the location of each medical institution. We used the “by prefecture” totals for each medical practice in FY 2021. The total numbers of items related to psychiatric day care facilities (the total value of items are presented in Table 2) by prefecture was used (N=47). Based on the total population of each prefecture (as of October 1, 2021) published by the Ministry of Internal Affairs and Communications, we calculated the number per 1,000 people per year for each prefecture. A Shapiro-Wilk’s test was performed to check that the data did not follow a normal distribution and a Mann-Whitney U test was conducted to compare the number per 1,000 people per year of prefectures with (N=16) or without capital- and government-designated cities (i.e., those with 700,000 or more people) (N=31). A Shapiro-Wilk’s test was performed to ensure that the data followed a normal distribution. A Pearson’s correlation coefficient test was conducted to examine the correlation between the number of psychiatry beds per 100,000 people in the 2022 medical facilities survey [16] and the annual total number of psychiatric day care facilities used per 1,000 people for each prefecture.

Differences Between Age Groups

Age group analysis was based on the “by sex and age” totals for FY 2021. The NDB Open Data were disaggregated by five-year increments. Owing to low numbers of patients under 15 years of age, interpreting the national trend would have been difficult as a limited number of facilities treated this age group; therefore, data for this age group were not tabulated.

Based on the total population by sex and age (as of October 1) published by the Ministry of Internal Affairs and Communications [17], we calculated the annual numbers of psychiatric day care facilities usage (the total value of items are presented in Table 2) per 1,000 people for each age group.

A Spearman’s rank correlation coefficient analysis was conducted to explore the association between the number of psychiatric day care facilities used per 1,000 people and age.

As individuals aged 65 and above are eligible for Long-Term Care Insurance, the data were classified into two groups: those under 65 (N=10) and those 65 and older (N=6). We then examined the association between age and each of these two groups.

Additionally, to examine the characteristics of the age group for each medical practice, we classified the data into age groups by 15-year increments (19 years and younger, 20-34, 35-49, 50-64, 65-74, and 75 years and older). The frequency of usage per 1,000 people was calculated for each group.

Sex Differences

The analysis was based on the “by sex and age” totals for FY 2017-2021. We calculated the total number of psychiatric day care facilities used (total value of items in Table 2) annually, segregated by sex, for patients aged 15 years and older. A Shapiro-Wilk’s test was performed to ensure that the data followed a normal distribution, and an unpaired t-test was conducted to compare the total annual visits (FY 2017-2021) by sex (Male: N=5, Female: N=5). All medical items related to psychiatric day care facilities were calculated based on FY 2021 data.

Statistical analysis

All statistical analyses were performed using IBM SPSS Statistics 29.0 (IBM Corp., Armonk, NY, USA). P-values were two-tailed, and values <0.05 were considered significant.

Ethical considerations

This study analyzed the data that are publicly available from the MHLW and the Ministry of Internal Affairs and Communications and did not deal with individual patient data. Therefore, there were no ethical issues.

## Results

Changes by fiscal year

The overall number of psychiatric day care facilities use exhibited a decreasing trend. The number of psychiatric short care use showed an upward trend through FY 2021. The number of psychiatric day-night care (>3 years / >3 days per week) use showed an increasing trend from FY 2020. The other categories exhibited a downward trend (Table 2).

**Figure 1:**
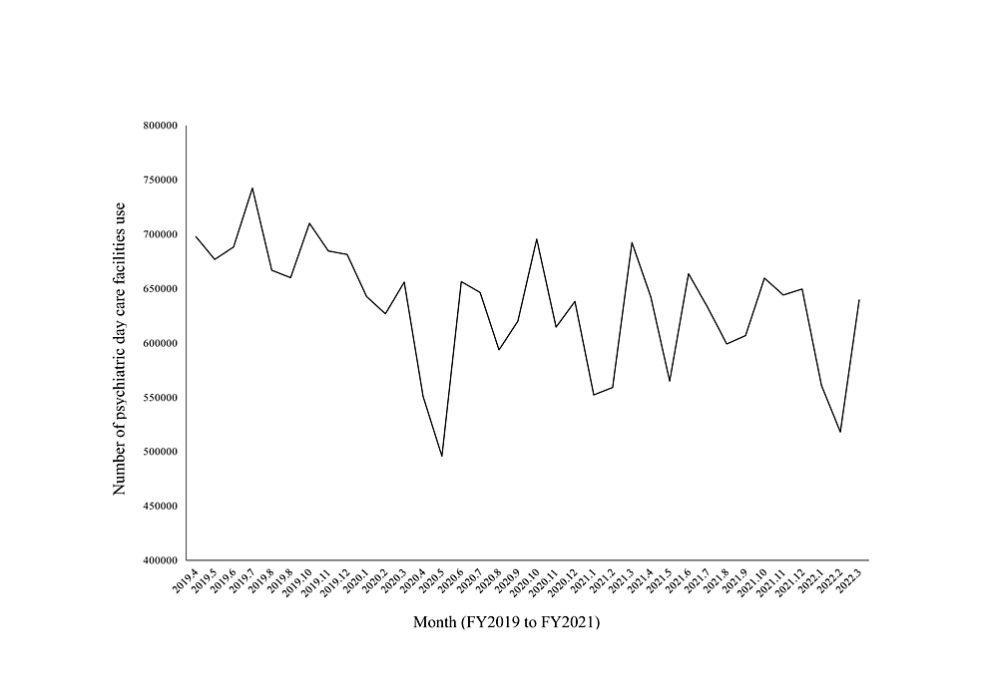
Total number of psychiatric day care facilities use by months (FY2019 to FY2021) (times) FY: fiscal year

The monthly data indicated a significant decrease in the total number of psychiatric day care facilities usage during periods of emergency declarations (April-May 2020, January-March 2021, April-June 2021, July-September 2021) and priority preventative measures (April-September 2021, January-March 2022) (p=0.02; Figure 1; Table 3).

**Table 3:** The monthly data of the number of psychiatric day care facilities use (FY2020-2021) (times) P: Mann-Whitney U test, *: p < 0.05, FY: fiscal year

	In months during periods of emergency declarations and priority preventive measures (N=14)	In months when they were not (N=10)	P	Cohen’s d
The number of psychiatric day care facilities use (Mean ± SD)	591385.4 ± 57321.1	641839.8 ± 27894.1	0.02^*^	1.06

Regional differences

**Figure 2:**
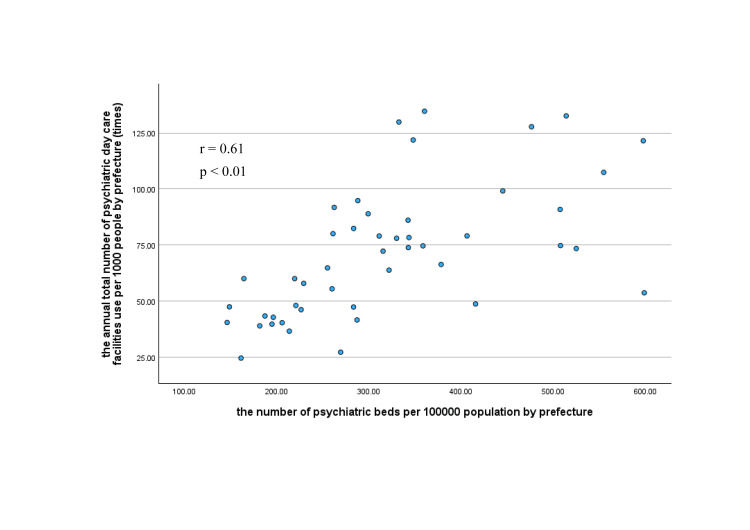
Correlation between the annual total number of psychiatric day care facilities use per 1000 people by prefecture and the number of psychiatric beds per 100000 population by prefecture r: Pearson’s correlation coefficient

The annual total number of psychiatric day care facilities use per 1,000 people, was the largest (134.7) in Okinawa Prefecture and the smallest (24.6) in Shiga Prefecture (Figure 2), with the former being 5.5 times the latter. The total annual usage of psychiatric day care facilities per 1,000 people was significantly lower in prefectures with capital- and government-designated cities than in other prefectures (p<0.01; Table 4). There was a significant positive correlation between the annual total number of psychiatric day care facilities per 1,000 people by prefecture and the number of psychiatric beds per 100,000 population by prefecture (r=0.61, p<0.01).

**Table 4:** The number of psychiatric day care facilities use per 1,000 people in prefectures with capital- and government-designed cities and other prefectures (times) P: Mann-Whitney U test, **: p < 0.01

	Prefectures with capital- and government-designed cities (N=16)	Other prefectures (N=31)	P	Cohen’s d
The number of psychiatric day care facilities use per 1,000 people (Mean ± SD)	51.6 ± 18.1	82.0 ± 28.8	<0.01^**^	1.18

Differences between age groups

**Figure 3:**
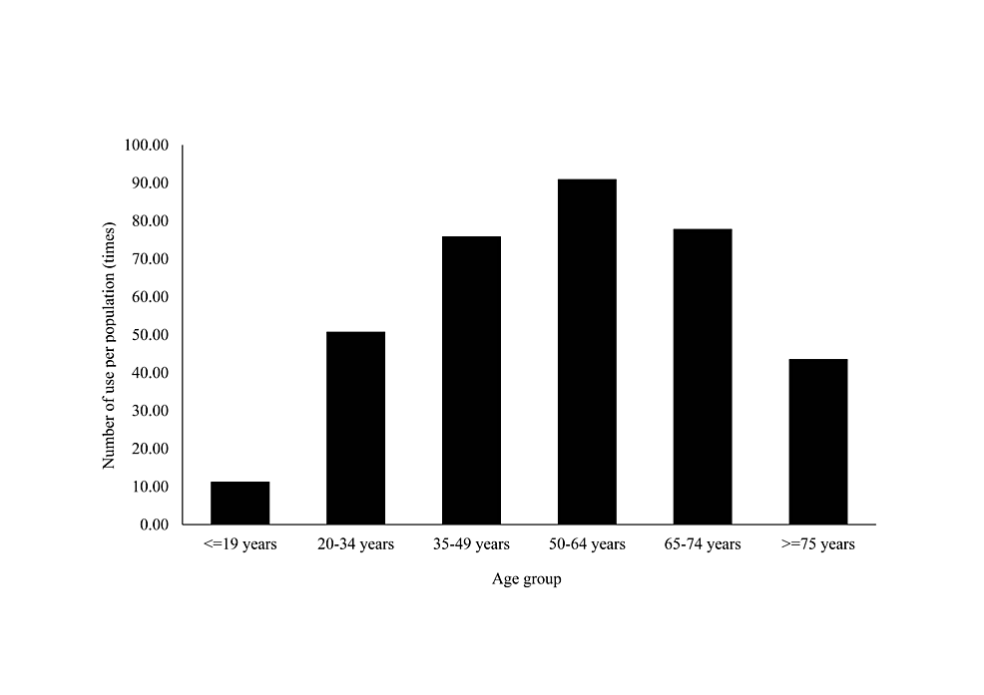
Total number of psychiatric day care facilities use per 1000 population for each age group (FY2021) (times) FY: fiscal year

The numbers per 1,000 people were the highest for all items in the 50-64 age group, with slightly lower for 15-19 and 20-34 age groups (Figure 3).

A significant positive correlation was found between age and the number of psychiatric day care facilities usage per population for those under 65 years of age (ρ=0.99, p<0.01). In contrast, there was a significant negative correlation between age and the number of psychiatric day care facilities usage per population for those 65 and older (ρ=-0.94, p<0.01; Table 5).

**Table 5:** Association between the number of psychiatric day care facilities use per 1,000 people and age group (by five years) P: Spearman’s rank correlation coefficient, **: p < 0.01

	ρ	P
Under 65 years of age (N=10)	0.99	<0.01^**^
65 years old and over (N=6)	-0.94	<0.01^**^

By item, psychiatric short care was the highest in the 35-49 age group, psychiatric day and night cares in the 50-64 age group, and day (>3 years / > 3 days per week) and day-night cares in the 65-74 age group (Table 6).

**Table 6:** Number of use per 1000 populations for each group (FY 2021) (times) FY: fiscal year

	<=19years	20〜34years	35〜49years	50〜64years	65〜74years	>=75years
Psychiatric short care(small)	1.38	3.95	4.00	3.06	1.27	0.66
Psychiatric short care(large)	2.60	10.16	12.12	10.01	4.58	1.47
Psychiatric day care(small)	0.74	4.82	8.22	9.79	7.70	0.00
Psychiatric day care(large)	5.01	26.21	39.92	47.90	41.03	24.30
Psychiatric day care（>3year / >3days per week）(small)	0.00	0.07	0.34	0.47	0.44	0.18
Psychiatric day care（>3year / >3days per week）(large)	0.06	0.77	2.12	2.46	2.50	1.75
Psychiatric night care	0.36	0.71	1.12	1.29	0.43	0.05
Psychiatric night care（>3year / >3days per week）	0.01	0.04	0.07	0.12	0.04	0.00
Psychiatric day-night care	0.97	3.75	7.39	14.76	18.41	10.38
Psychiatric day-night care（>3year / >3days per week）	0.04	0.19	0.51	1.05	1.37	0.84

Sex difference

Data from FY 2017-2021 showed that men used psychiatric day care facilities more frequently than women (p<0.01; Table 7). For the number of psychiatric day care facilities used in FY 2021, all items were significantly higher for men than women (Table 8).

**Table 7:** The annual total number of psychiatric day care facilities use by sex (FY2017-2021) P: Unpaired t-test, **: p<0.01, FY: fiscal year

	Male (N=5)	Female (N=5)	P	Cohen’s d
The annual total number of psychiatric day care facilities use (Mean ± SD)	4685807.0 ± 350106.3	3186590.6 ± 168370.4	<0.01^**^	5.46

**Table 8:** Numbers of use by sex (FY2021) (times) FY: fiscal year

Medical practice	Male	Female
Total number of psychiatric day care facilities use	4308301	3041641
Psychiatirc short care（small）	160613	133255
Psychiatirc short care（large）	504914	363249
Psychiatirc day care（small）	420265	330409
Psychiatirc day care（large）	2263451	1621494
Psychiatirc day care（small）（>3year / >3days per week）	19222	13520
Psychiatirc day care（large）（>3year / >3days per week）	122720	82601
Psychiatric night care	57691	26277
Psychiatric night care（>3year / >3days per week）	4664	1463
Psychiatric day-night care	704036	437833
Psychiatric day-night care（>3year / >3days per week）	50725	31540

## Discussion

This study used NDB Open Data to analyze trends in the numbers of psychiatric day care facilities used in Japan, as well as regional, age, and sex differences.

The descriptive data for FY 2017-2021 showed the total number of psychiatric day care facilities used had been declining since FY 2020. The number of psychiatric day care facilities used was also significantly smaller in the months during the period of emergency declarations and priority preventative measures than in other months in FY 2020-2021. Previous studies outside Japan have reported that the number of services for outpatients with mental illness and rehabilitation needs, including psychiatric day care, has decreased since 2020 owing to the COVID-19 pandemic-related lockdown [18,19]. Our results were consistent with this, suggesting that the number had decreased due to effects such as staying at home and the operational scale of facilities; this trend continued in FY 2021.

In contrast, descriptive data showed that the numbers of psychiatric day-night care used (>3 years / >3 days per week) and short care used has increased since 2020. The increase in psychiatric short-care usage may imply that the utilization of psychiatric day care facilities reduced for reasons such as infection control. Moreover, the increase in psychiatric day-night care usage (>3 years / >3 days per week) might have occurred because the patients used psychiatric day care facilities for a longer period and frequently needed support. A previous study indicated that supporting patients with severe mental illness was difficult during the COVID-19 pandemic because of limited social networks [20]. Additionally, another qualitative study on patients with schizophrenia reported that outpatients could not visit hospitals and lost contact with mental healthcare professionals because of the COVID-19 pandemic [21]. Thus, patients with mental illnesses might have had difficulty receiving support during the pandemic. However, the increase in the number of psychiatric day-night care (>3 years / >3 days per week) used suggests that resources for support were available in psychiatric day care facilities during the COVID-19 pandemic. Therefore, it was considered that the increase in the number of times these medical practice items were used was observed.

Previous research has suggested regional differences in psychiatric medical systems [22]. However, no quantitative research has been conducted on regional differences in the operational status of psychiatric day care in Japan. In the NDB Open Data, the regional differences in the total number of psychiatric day care facilities use per 1,000 people increased 5.5 times, suggesting gaps in the operational status of psychiatric day care facilities among prefectures. Furthermore, prefectures with more psychiatric beds per 1,000 people had more psychiatric day care facilities used. Psychiatric beds per 1,000 people tends to concentrate in peripheral areas, particularly in the Kyushu and Shikoku coastal areas [23]. Japan has more psychiatric beds than any other OECD country and also has one of the longest psychiatric hospitalization periods. Historically, psychiatric day care facilities in Japan have been developed for patients who are hospitalized long-term after discharge from a psychiatric hospital. Therefore, the number of psychiatric beds had no small impact on regional differences in psychiatric day care use. Thus, even today, more psychiatric beds per 1,000 population in a region implies more availability of psychiatric day care facilities as a social resource of rehabilitation facilities for outpatients.

However, the usage numbers were significantly smaller in urban areas (prefectures with cities designed by the government). In addition to prefectures, government-designed cities are required to establish mental health and welfare centers. A well-functioning mental health center makes it easier to connect to resources other than psychiatric day care facilities, including disability welfare services, resulting in fewer psychiatric day care facilities used. Furthermore, Japan has enacted the Medical Expenses for Services and Support for Persons with Disabilities, a public financial support system for psychiatric outpatient treatment. Typically, co-payment for medical expenses accounts for 30% of the total; however, the Medical Expenses for Services and Supports for Persons with Disabilities system can be used to reduce out-of-pocket expenses for psychiatric outpatient visits and psychiatric day care facilities to 10% of the total cost. Previous research has suggested that this system can contribute to persistence with outpatient treatment [24]. Therefore, it was considered possible that this may have affected the number of psychiatric day care facilities used. In some cases, the local governments (e.g., Okinawa Prefecture [25] and Hiroshima City [26]) pay the co-payment portion of the Medical Expense for Services and Supports for Persons with Disabilities, resulting in regional differences in the operations related to psychiatric treatment. However, no reports have summarized the operations of local governments. Therefore, detailed research is required to analyze the relationship between regional differences in the operations of Medical Expense for Services and Support for Persons with Disabilities and the number of psychiatric day care facilities used. Additionally, it is possible that several factors not examined in this study may have influenced regional differences in the frequency of use. The prevalence of each mental disorder by region and differences in the level of acceptance of psychiatric care in each region were considered possible factors. Therefore, it is considered necessary to include these factors in further studies.

Regarding sex differences, the results showed that the numbers were significantly higher for men, which is more likely to be linked to the use of psychiatric day care facilities. However, previous research outside Japan has indicated that women are more willing to use mental health services [27], which contradicts our results. In contrast, previous research investigating the recognition of mental health services in Japan showed that men are more willing to use mental health services because they are more likely to be actively involved in society and have more opportunities to learn about mental health [28]. For example, women in Japan are more likely to limit their social participation due to childbirth and childcare. This may be related to the low frequency of use of psychiatric day care. Thus, Japanese social and cultural backgrounds may influence sex differences in the use of psychiatric day care facilities. Accordingly, the need to build understanding and support systems to bridge the gender gap was considered.

Regarding age, under 65 years of age and younger age groups were less likely to use psychiatric day care facilities. A previous study has pointed out that younger age groups tend to have more negative feelings toward using mental health services [28]. Thus, differences in perceptions among various age groups may influence the use of psychiatric day care. It is considered necessary to analyze the needs of young people and consider how to run programs to promote youth participation in society and create an environment where young people can easily participate. Additionally, the proportion of middle-aged or older patients is higher than that of younger patients [29]. Therefore, the use of psychiatric day care facilities as a supportive institution for middle-aged and older patients discharged from hospital might have led to an increase in psychiatric day care facility users. Moreover, an increasing number of older parents need to provide financial and emotional support to their middle-aged and older children who are mentally ill or withdrawn. This is a social issue in Japan, often highlighted as the “8050 issue” [30]. The higher numbers for middle-aged and older patients may reflect the fact that psychiatric day care promotes independence among middle-aged and older patients. In contrast, older age groups (65 years old and older) were less likely to use psychiatric day care facilities, possibly because they were using other services, such as long-term care insurance services, instead of psychiatric day care facilities.

This study had some limitations. Items with less than 10 uses may lead to under-reporting and bias in areas with small populations or infrequent use of certain medical practices. Furthermore, there is the influence of other unmeasured confounding factors. NDB Open Data covers the number of calculations for the entire country; however, the data are solely based on the number of psychiatric day care facilities used. Therefore, they do not include information regarding individual patient characteristics such as sociodemographic data, diagnosis, symptoms, duration of illness, treatment history, and details of treatment. Additionally, this study included analyses where only descriptive statistics were conducted. Furthermore, it may not reflect the total number of psychiatric day care users in Japan. Methodological limitations also exist, such as small sample sizes in statistical analyses and low precision in estimating effect sizes. Therefore, the results should be interpreted with caution. In the future, it will be necessary to clarify the actual situation in more detail using large-scale medical databases, such as the NDB, as well as nationwide patient surveys that reflect patient characteristics.

## Conclusions

This is the first study to quantitatively examine the operational status of psychiatric day care facilities across Japan using NDB Open Data. The results assume that the operational status of psychiatric day care facilities were affected by regional differences in psychiatric beds per population and operational status related to social and financial support for patients with mental illnesses. To improve regional differences, it is necessary to consider support for healthcare costs and the operation of facilities tailored to local needs. Additionally, this study showed that while psychiatric day care facilities can be utilized for support in emergency situations to live a fulfilling life in the community despite mental disorders, their utilization differs according to sex and age. It is necessary to consider approaches for people with certain attributes who are less likely to use psychiatric day care facilities. Specifically, there is a need to create systems and environments that make it easier for young people and women to use the facilities, and to prepare programs that facilitate their participation. Regarding regional differences, it may also be necessary to consider expanding the number of facilities and support for medical costs. For patients who are difficult to reach for the provision of psychiatric day care support, it is necessary to develop an operational structure and program based on the current conditions of available facilities and the community.
